# Case Report: Successful laparoscopic approach for the management of a voluminous left paratubal cyst: two cases and a literature review

**DOI:** 10.3389/fsurg.2026.1859811

**Published:** 2026-07-08

**Authors:** Asma Sghaier, Sabri Youssef

**Affiliations:** 1 Department of Digestive and Visceral Surgery, Hospital of Farhat Hached of Sousse, Sousse, Tunisia; 2 Faculty of Medicine of Sousse-University of Sousse, Sousse, Tunisia

**Keywords:** abdominal mass, adnexal cyst, laparoscopy, radiology, surgery

## Abstract

**Introduction:**

Paratubal or adnexal cysts develop in the broad ligament of the uterus. They are regarded as giant when the 150 mm threshold is reached. Clinical features and presenting symptoms include either the effects of compression of the adjacent organs or complications. Surgical resection of these cystic masses is required for diagnosis and treatment.

**Case presentation:**

We report the cases of two young women who complained of pelvic pain and progressive abdominal distension. Investigations indicated that paratubal cysts were compressing their adjacent structures. The medical staff's decision was to operate on these patients using a laparoscopic approach to provide histological confirmation and treat their symptoms. This decision was taken on the basis of the lack of morphological signs of benignity. Thus, the patients were managed via a laparoscopic approach to provide histological confirmation and symptom relief. The procedure was performed under a pneumoperitoneum of 12 mmHg with a specific strategy to prevent spillage.

**Discussion:**

A laparoscopic approach in the management of large paratubal cysts appears to be safe and feasible. However, expertise is required for the successful removal of the mass. The first challenge is to avoid spilling the cyst’s contents, as there is no evidence of benignity, and the second is to preserve the ovaries and adnexa for fertility.

**Conclusion:**

Cystic paratubal masses require comprehensive investigations to determine their nature and origin. However, only surgical resection can confirm the diagnosis, as it offers the possibility of histological confirmation. Moreover, a laparoscopic approach is also useful for treating the patient’s symptoms. It is important to note, however, that the size of the cyst could limit the use of this approach; therefore, expertise is required.

## Introduction

Paratubal cysts are generally small; however, a few cases of large paratubal cysts exist. Surgical lesions of the adnexa in adolescents cover a variety of ovarian and para-ovarian lesions, including benign functional cysts, malignant neoplasms, and torsion of the ovary and/or fallopian tube (1). Patients may complain of pelvic masses, acute abdomen, and, in some cases, may present with premature puberty or virilization (2). Acute pain could be the result of cystic bleeding or disruption, adnexal twisting or infection, or compression of adjacent organs. Furthermore, paratubal cysts are vestiges of Müllerian eminences or eminences of mesothelial origin. They are incidental findings during an ultrasound scan or an operation and are seldom symptomatic. They typically measure less than 8 cm, but large cysts have been discovered and are challenging to manage (3). Surgical excision is indicated in all cases with large masses because there is a risk of ovarian torsion and necrosis. The best surgical approach is minimally invasive surgery, laparoscopy, or, in recent years, robotic surgery. The laparoscopic approach is the gold standard for the treatment of benign adnexal masses. However, for voluminous cysts, due to the possibility of malignancy and space restrictions, many surgeons opt for a laparotomy instead of laparoscopy (4). The use of sealing devices is essential for dissection to avoid bleeding. Moreover, the use of metal clips to control vessel dissections may make the procedure easier for surgeons. Regarding cysts in adolescents, the initial approach should always be conservative (5, 6). In this case report, we report two patients who complained of pelvic pain, for whom successive investigations had concluded that they had voluminous left paratubal cystic masses. The masses were 220 and 280 mm in diameter on their largest axis, respectively. The patients underwent laparoscopic surgery with a three-trocar approach. This procedure was accomplished without cyst spillage. These cases are reported in line with the Surgical CAse REport (SCARE) criteria (7).

## Case presentations

We report two cases of voluminous left paratubal cysts successfully managed with a laparoscopic approach in the Department of Surgery, Hospital of Farhat Hached, Sousse, Tunisia.

### Case 1 (2023)

The patient was 26 years old, had no previous medical or surgical history, and presented with pelvic pain. She was married, had undergone no abortions, and her menstrual cycles were regular. During the medical interview, the patient reported that she had had her menarche when she was 12 years old. The patient had been complaining of pelvic pain for 2 months, with a sense of heaviness associated with the symptoms. She had no urinary complaints or menstrual cycle disorder. An abdominal examination revealed a soft, painless, distensible abdomen, with evidence of a firm, painless, and mobile mass in the hypogastrium. The mass was approximately 270 mm in diameter; there were no obvious inflammatory features. The hernial orifices were free, and the rectal examination revealed no detectable abnormalities. The gynecological examination was normal. To determine malignancy risk, the preoperative assessment included CA-125 and carcinoembryonic antigen (CEA) markers, which were normal. A pelvic ultrasound coupled with an endo-vaginal ultrasound showed no abnormalities of the genital tract but revealed a left latero-uterine anechoic cystic mass measuring 250 mm on its long axis, without identifying any intracystic vegetations. The ovaries were of normal dimensions and ultrasound appearance.

This finding was further complemented by an abdominopelvic computed tomography (CT) scan, which was performed after the administration of intravenous contrast material and described the same cystic lesion adjoining the left paratubal area. We described the mass as follows: a formation of homogeneous liquid density, unilocular, with a fine median surface, above the bladder, displacing the uterus posteriorily and extending anteriorly to contact abdominal Wall it measured 270 mm × 132 mm × 65 mm in the axial plane and extended over 28 mm in height. The ovaries were visualized and follicular.

**Figure 1:**
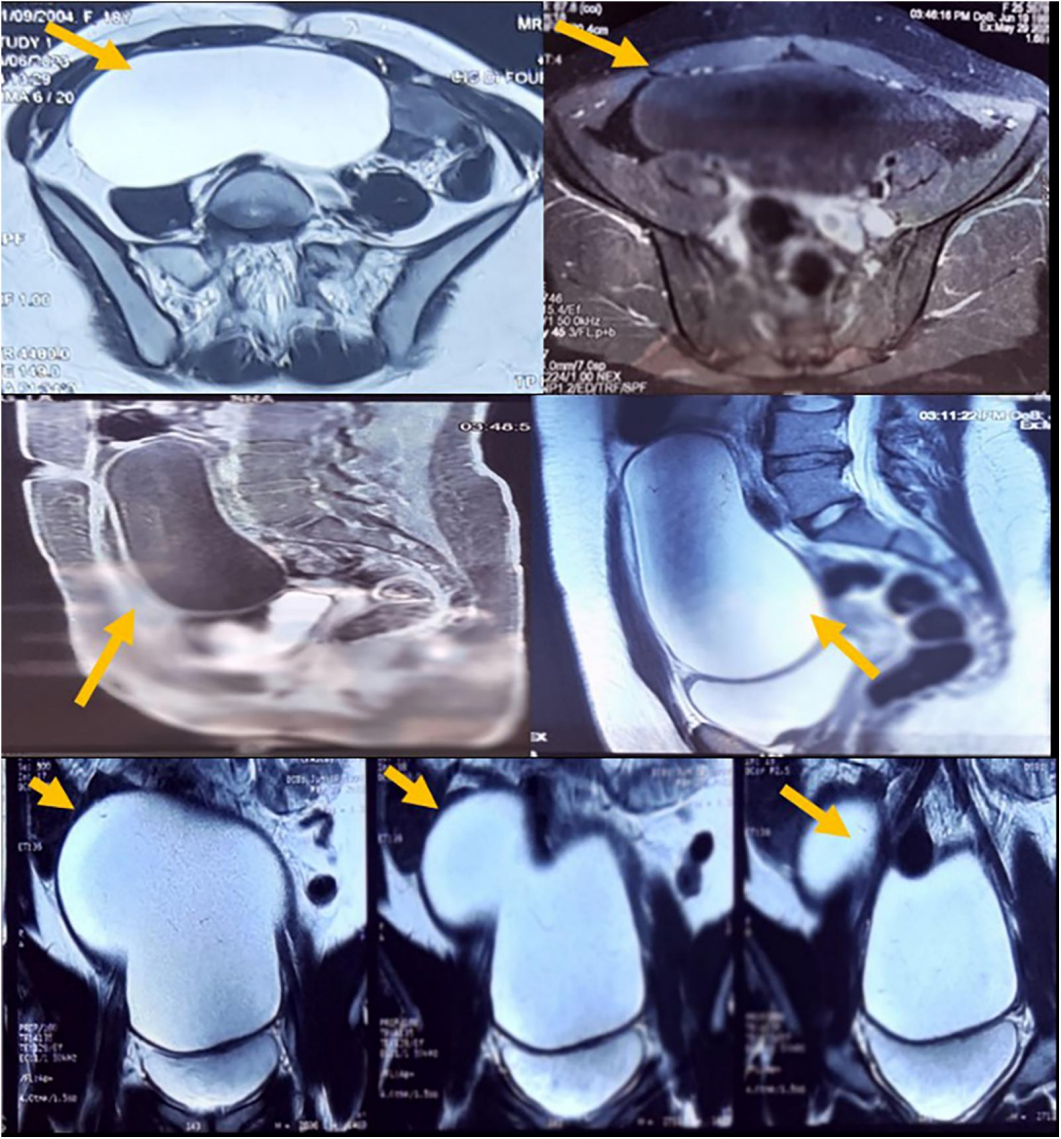
T2-weighted MRI highlights the relationship between the 280 mm cyst and the displaced uterus (yellow arrows indicate the limits of the large, unilocular cystic mass).

Magnetic resonance imaging (MRI) revealed a left anterior cystic mass centered on the abdomen that was oval and multilocular, hypointense on T1 and homogeneously hyperintense on T2, without diffusion restriction, and measuring 280 mm × 132 mm × 90 mm. It had a fine wall with no endocavitary buds or pathological enhancement. The uterus and left ovary are displaced downward, and the left ovary was displaced outward, while no other abnormalities were detected. All the morphological investigations concluded that the cyst was a left para-adnexal cyst with no prior radiological evidence of malignancy (Figure 1).

Our differential diagnosis included the following: giant ovarian serous cystadenoma: excluded by radiological identification of normal ipsilateral ovaries separate from the mass; hydrosalpinx: excluded due to the lack of “tubular” or “waist” signs on MRI; and mesenteric cyst: excluded by a lack of bowel wall involvement and the pelvic origin of the mass.

Preoperative risk stratification: Preoperative management focused on differentiating a benign paratubal cyst from a malignant adnexal tumor or functional ovarian cyst.

Risk scores: We utilized the International Ovarian Tumor Analysis (IOTA) Simple Rules system and ultrasound; the mass was classified as benign due to its unilocular nature, absence of solid components, and a lack of blood flow on Doppler ultrasound (8).

The decision of the medical staff was to operate on the patient via laparoscopy for both diagnostic and therapeutic purposes. A possible ovariectomy if the mass proved to be of ovarian origin was discussed with the patient, and the therapeutic options were also discussed at length. Written consent, signed by the patient, was obtained prior to surgery. She was admitted on 11 March and underwent laparoscopic surgery on 12 March. The patient was placed in the dorsal decubitus position with her legs together and her left arm at her side. The procedure followed a standardized three-trocar laparoscopic approach. A pneumoperitoneum was established using the Veress technique at the left hypochondrium to prevent the mass from reaching the umbilicus. The first 10 mm trocar was introduced through the left hypochondrium to accommodate the 30° optical camera.

### Surgical procedure and stepwise description

**Figure 2:**
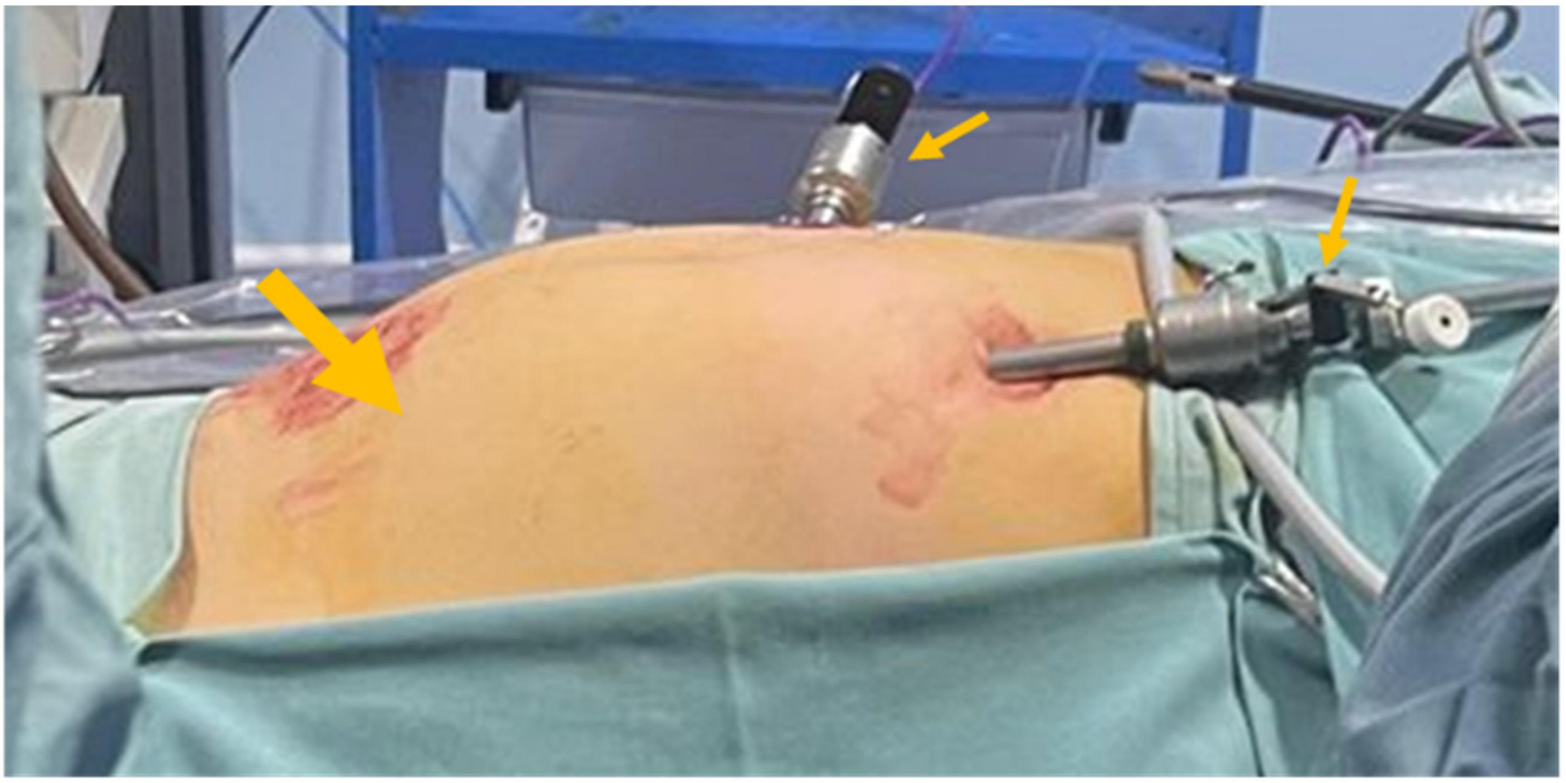
Placement of trocars.

**Figure 3:**
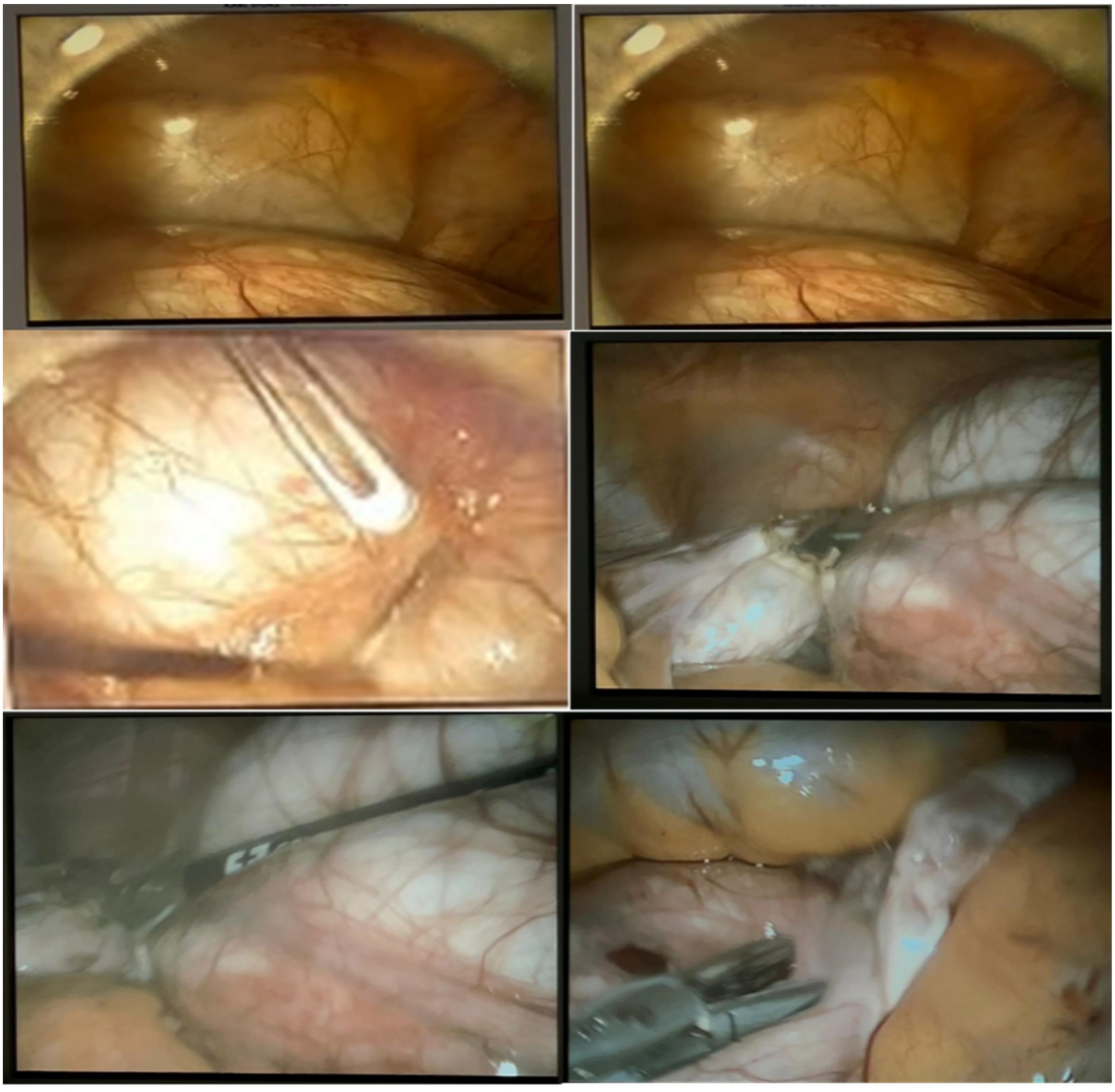
Operative views during laparoscopy.

The surgical procedure was performed under general anesthesia with the patient in the Trendelenburg position (15°–20° tilt) to allow for cephalad displacement of the bowel. Due to the voluminous nature of the cyst, which reached the umbilicus, a closed entry technique using a Veress needle was performed at Palmer's point (left hypochondrium, 3 cm below the costal margin in the midclavicular line). This site was selected to ensure a safe distance from the superior edge of the cystic mass. A pneumoperitoneum was established and maintained at a controlled pressure of 12 mmHg. A 10-mm optical trocar was inserted into the left hypochondrium to accommodate the 30° laparoscope. Under direct vision, two additional trocars were placed: a 10-mm operating trocar in the right hypochondrium and a 5 mm accessory trocar in the suprapubic region for retraction and exposure (Figure 2). Intraoperatively, the cyst was manipulated using atraumatic graspers to prevent rupture. Exploration confirmed the absence of ascites. Adhesions between the cyst wall and the mesosalpinx were dissected using advanced bipolar thermofusion pliers (e.g., LigaSure or similar), ensuring meticulous hemostasis. To maintain oncological safety, an endobag was introduced. The intact cyst was placed inside the bag intra-abdominally. The neck of the bag was then exteriorized through the 10-mm trocar site. Only then was percutaneous aspiration of the cyst’s contents performed within the bag at the skin level. This controlled drainage reduced the cyst’s volume, allowing for safe extraction through the small incision without intraperitoneal contamination (Figure 3).

**Figure 4:**
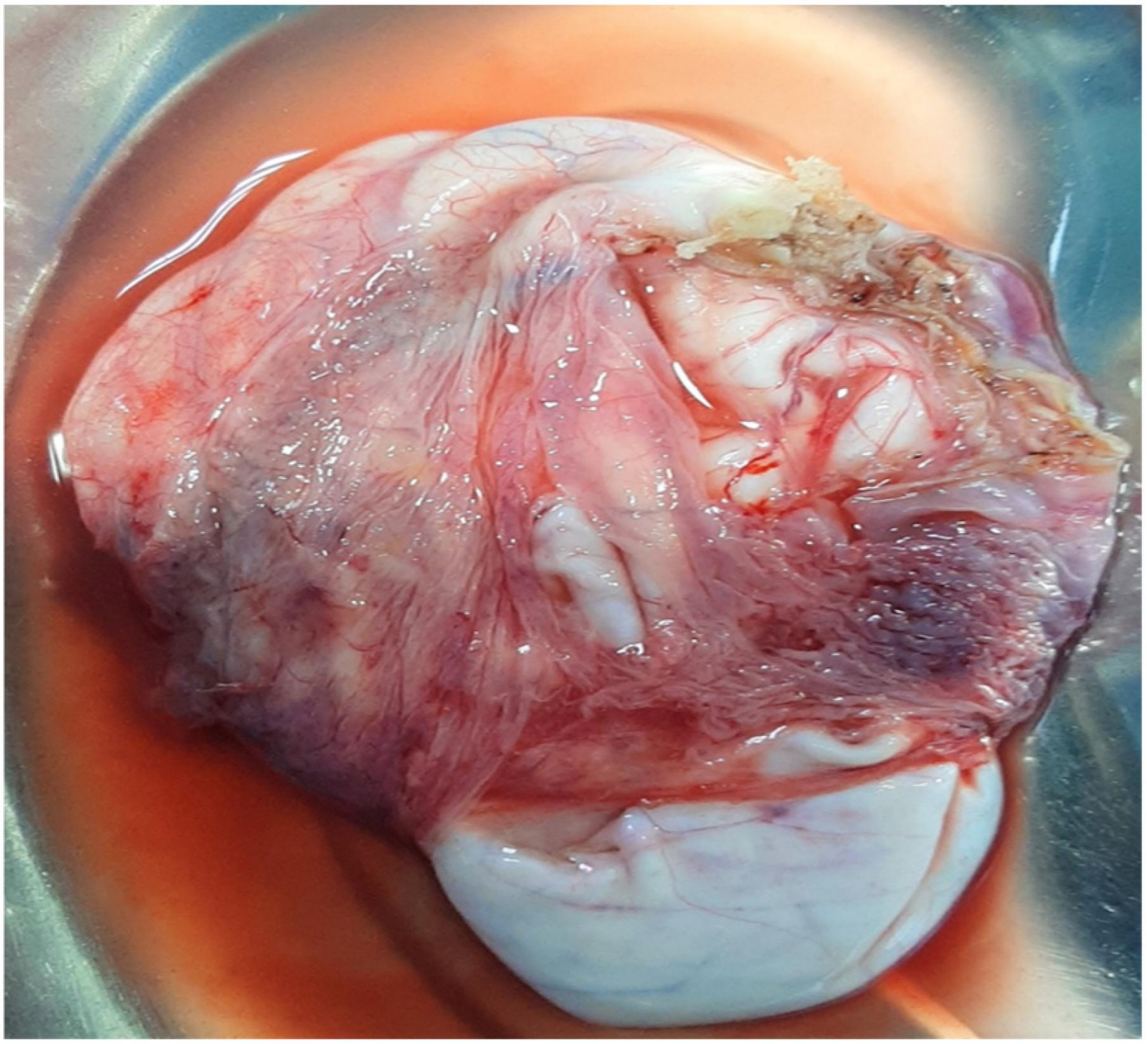
Surgical specimen.

The cystic mass was then placed into a bag. The cyst was extracted via a small enlargement of the suprapubic trocar orifice. For easier retrieval of the cyst, its contents were aspirated as soon as the cutaneous incision was in contact with the cystic mass. Macroscopic examination after extraction of the cyst revealed serous water-like contents, a thin wall, and no vegetation within the cyst (Figure 4).

The postoperative course was good, and the patient was discharged from the hospital the following day. At a scheduled follow-up visit 3 months later, she remained well with no further abdominal symptoms. Histological examination postoperatively confirmed a benign serous cyst with no signs of malignancy.

The patient's episode of care followed an efficient clinical timeline, beginning with her admission on 11 March. She underwent the laparoscopic surgical procedure the following day, 12 March. Following a successful recovery with a good postoperative course, the patient was discharged on 13 March, the first postoperative day.

### Case 2

The second patient was 18 years old, with no notable medical or surgical history and no previous gynecological history, in particular no menarche disturbances, and had presented with abdominal pain associated with constipation. The patient had no complaints apart from the associated sensation of heaviness. The gynecological examination was normal. On examination, there was a large pelvic mass, the upper edge of which extended beyond the umbilicus. A physical examination revealed an abdominopelvic mass with well-defined limits, the upper border extending approximately three finger-widths below the xiphoid process. The mass was mobile, painless, and firm to palpation. There were no signs of inflammation. A rectal examination revealed no detectable abnormalities. Blood tests were normal, including tumor markers (CEA and CA-125). Alpha-fetoprotein and beta-human chorionic gonadotropin were considered to exclude germ cell tumors, though the clear liquid signal on T2-weighted MRI strongly suggested a serous paratubal origin. Colonoscopy was normal. We completed a pelvic ultrasound, combined with an endovaginal ultrasound, which showed a large anechoic cystic mass measuring 200 mm. The ovaries and uterus were of normal size and morphology. This was complemented by magnetic resonance imaging, which described the mass as follows: a cystic formation measuring 220 mm × 180 mm × 70 mm, independent of the ovaries, with a thin wall, no septum or vegetations, a homogeneous signal on all sequences, hypointense on T1, hyperintense on T2, clear liquid type, and no pathological enhancement after a gadolinium injection. The examination revealed no further abnormalities. The patient was admitted on 20 January 2023 and underwent surgery on 24 January 2023.

The patient was placed in the Trendelenburg position. A 10-mm optical trocar was inserted into the left hypochondrium (Palmer's point) to avoid directly puncturing the giant cyst. Two additional trocars (10 and 5 mm) were placed under direct vision. The cyst was dissected using advanced bipolar thermofusion pliers. To prevent spillage, the intact cyst was placed in a retrieval endobag. Controlled aspiration of the liquid content was performed only after the bag was exteriorized at the skin level, ensuring no intra-abdominal leakage.

## Results and follow-up

The operative time averaged 95 min with minimal blood loss (<50 mL). The histological examination confirmed a benign serous cyst lined with a simple cuboidal epithelium, with no signs of borderline malignancy or atypia. Both patients were discharged on postoperative day 1. At the 3-month follow-up, both were asymptomatic with normal ultrasound findings.

## Discussion

Paratubal cysts develop in the broad ligament of the uterus. They are regarded as giant when the 150 mm threshold is reached. Clinical features and presenting symptoms are due to either the effects of compression of adjacent organs or complications. Abdominal ultrasound, CT, and MRI are helpful diagnostic imaging aids; however, the exact origin of these large cysts is most often only revealed by surgical exploration (9). Paratubal cysts and paraovarian cysts are both situated in the mesosalpinx of the broad ligament (10). They may arise from the mesothelium, parametonephric tissue, or remnants of the mesonephric vestiges (11). These masses represent approximately 10% of all adnexal masses (12). They are more frequent in the third or fourth decade of life, with a prevalence among postmenopausal women of 6.25%, while only 4% of cases occur in adolescence (13, 14). In both of our cases, the patients were young. Their main complaint was pelvic pain with a feeling of heaviness. This complaint has been reported in the majority of studies published in the literature (15).

The symptoms of paratubal cysts are non-specific. Small cysts are asymptomatic and are typically discovered during radiologic investigations or surgery for abdominopelvic pathologies. In large cystic masses, the symptoms are also non-specific, such as abdomino-pelvic pain, an increase in abdominal volume, or a feeling of heaviness in the pelvis. These symptoms are the result of compression of adjacent organs. Therefore, urinary signs, such as dysuria, pollakiuria, and hydronephrosis, or compression of the digestive tract may also be observed. In this latter situation, the most common manifestation is constipation or vomiting (15). Among those with giant cysts, genital disorders such as menstrual irregularities, dyspareunia, and, in a few cases, uterine prolapse have been observed. Pelvic ultrasound and, when appropriate, pelvic MRI are the most effective imaging procedures for studying these masses, identifying their nature, origin, and dimensions, and detecting any complications (16).

The suitable treatment for a large cyst should be surgical resection; however, currently, there is no recommendation on whether this should be conducted using a laparoscopic or open approach. Although a laparotomy is often preferred for voluminous cysts to prevent malignancy spillage, we opted for laparoscopy due to the benign radiological features (fine walls and no septa) and the desire for a rapid recovery in these young patients. Generally, the main factors limiting the use of laparoscopy are the large size of the cyst, evidence of malignancy, or a lack of surgical expertise (17). A systematic search was conducted in PubMed and Google Scholar using the keywords “Giant Paratubal Cyst” and “Laparoscopy” for the period 2015–2022 (Table 1).

**Table 1:** Successful laparoscopic removal of large paratubal cysts in the literature.

Author(s)	Year	Age	Size (mm)	Clinical feature	Surgical approach	Histological feature
Asare et al. (18)	2015	19	270	Pelvic mass	Laparoscopy	Paratubal cyst
Shah et al. (19)	2016	16	260	Pelvic pain	Laparoscopy	Paratubal cyst
Tsuji et al. (20)	2017	25	340	Pregnancy	Laparoscopy	Paratubal cyst
Mărginean et al. (21)	2018	15	170	Abdominal mass	Laparoscopy	Serous cyst
Skaff et al. (22)	2019	31	360	Abdominal mass, pelvic pain	Laparoscopy	Serous Paratubal cyst
Atileh et al. (23)	2020	32	400	Abdominal mass	Laparoscopy	Serous cystadenoma
Alpendre et al. (17)	2020	31	250	Pelvic pain, constipation, vomiting	laparoscopy	Serous papillary cystadenoma
Bhansakarya and Subedi (3)	2020	25	270	Abdominal mass, pelvic pain	Laparoscopy	Serous cystadenoma
Čančar et al. (24)	2021	26	580	Pelvic pain, dysuria, constipation, uterine prolapse	Laparoscopy	Simple serous cyst
Kiran et al. (11)	2021	13	230	Pelvic mass, pelvic pain, metrorrhagia	Laparoscopy	Serous cystadenoma
Tjokroprawiro (9)	2021	30	223	Pelvic pain, abdominal mass	Laparoscopy	Serous benign cyst
Shamail et al. (25)	2022	19	300	Metrorrhagia, pelvic pain, abdominal mass	Laparoscopy	Serous benign cyst
Our cases	2023	26 18	280 220	Pelvic pain, abdominal mass	Laparoscopy	Paratubal serous cyst

The main challenge remains the risk of content spillage. If malignancy is suspected (e.g., presence of vegetations on MRI or elevated CA-125 levels), laparoscopy should be approached with extreme caution or converted to laparotomy. In our cases, the absence of radiological “red flags” and the use of the endobag extraction technique allowed for a safe, minimally invasive approach. A few large paratubal cysts treated laparoscopically have been reported in the literature in the last decade (Table 1). Aspiration of the cyst’s contents was required for diagnosis and to provide better and easier dissection. The main goal of the treatment should be to spare the ovaries and adnexa to preserve fertility. Nevertheless, the removal of a large cyst sometimes requires associated tubal excision or ovarian sacrifice.

### Critical comparison with the literature

While laparotomy is traditionally favored for cysts exceeding 10–15 cm to avoid rupture, our experience aligns with recent studies by Skaff et al. and Tjokroprawiro, who suggested that the laparoscopic approach is not only feasible but potentially superior in terms of recovery and cosmetic outcomes for young patients. However, unlike some authors who advocate for preoperative percutaneous aspiration to create a working space, we argue that intra-abdominal bag containment before aspiration is a safer “oncological” safeguard against potential borderline malignancy that may be missed by imaging. The laparoscopic approach is trending toward becoming the gold standard for the management of large paratubal cysts, although it remains controversial. An increase in the size of the cyst appears to be proportionally associated with the risk of rupture and spillage of its contents. This can have deleterious consequences if the cyst is proven to be malignant, especially as there is no definitive diagnosis before surgery. This is the reason for requiring extreme caution and dexterity when manipulating the cyst. Aspiration of the cyst’s contents is controversial, as it is always uncertain whether the cyst is benign preoperatively (15). The surgeon's experience is therefore of the utmost importance to the success of this approach. A crucial requirement of this approach is to preserve the ovarian reserve and fertility by maintaining the ovaries and adnexa. This is one of the dilemmas of this surgery when the cyst is giant or complicated by rupture, torsion, or necrosis (5).

In this case report, we have illustrated the laparoscopic management of two large para-tubal cysts in two different patients. This procedure was successfully completed without ovarian or adnexal sacrifice and without rupture or spillage of contents. Our approach is unique because it demonstrates that even with a 280-mm mass, a three-trocar strategy is feasible without prior percutaneous aspiration, which remains controversial due to the potential for seeding.

Despite the success in these cases, the following limitations must be acknowledged: restricted working space: Giant cysts significantly limit the “triangulation” of laparoscopic instruments, requiring high surgical dexterity and often unconventional trocar placement (e.g., Palmer's point); tactile feedback: the loss of haptic feedback in laparoscopy makes it harder to distinguish between simple inflammatory adhesions and invasive tissue compared to open surgery; and sampling error: while we utilized MRI and markers, the lack of frozen section availability in all centers remains a limitation when choosing a minimally invasive route for large masses.

The feasibility and superiority of this approach for the treatment of large paratubal cysts are still debated, despite the numerous advantages of this approach. More advanced studies involving larger cohorts and randomized controlled trials are required to establish recommendations with a high level of scientific evidence regarding the management of large paratubal cystic masses using the laparoscopic approach.

## Conclusions

Paratubal cysts are a fairly common pathology. Surgical resection is required when they increase in size, and more so when they are voluminous. Surgery provides histological evidence and radical treatment of the cyst. The dilemma in the management of these cysts is the choice of approach. This question is being raised even more with the current trend toward laparoscopic techniques. However, laparoscopic surgery for paratubal cysts is challenging on several levels. The first is that no evidence of the benignity of the cyst is obtained preoperatively. The second is the need to remove the cyst without ovarian and adnexal sacrifice to guarantee preservation of fertility. The third challenge is to successfully complete the procedure without adverse events despite the geant size of the cyst, ensuring definitive histological confirmation and symtom relief. While feasible, laparoscopy for giant cysts requires surgical expertise and strict adherence to oncological safety principles, such as the use of retrieval bags to prevent peritoneal seeding.

The laparoscopic approach has been proven effective for small cysts and appears to be feasible and safe for large cysts. Nevertheless, further research must be conducted to provide high-level scientific recommendations.

## Data Availability

The raw data supporting the conclusions of this article will be made available by the authors, without undue reservation.
